# Mapping Handgrip Strength Research in Sports Performance: A Bibliometric Review of Applications, Trends, and Future Directions

**DOI:** 10.3390/sports14030101

**Published:** 2026-03-04

**Authors:** Exal Garcia-Carrillo, Diana Salas-Gómez, Antonio Castillo-Paredes, Boryi A. Becerra-Patiño, Claudio Farías-Valenzuela, Guillermo Cortés-Roco, Miguel Alarcón-Rivera, Héctor Fuentes-Barría, Rodrigo Yáñez-Sepúlveda

**Affiliations:** 1 Department of Physical Activity Sciences, Faculty of Education Sciences, Universidad Católica del Maule, Talca 3480112, Chile; exal.garcia@gmail.com; 2 Department of Physical Activity Sciences, Universidad de Los Lagos, Osorno 5290000, Chile; 3 Social Impact and Innovation in Health (InHEALTH), Nursing and Occupational Therapy College, University of Extremadura, 10003 Cáceres, Spain; diana.salas.gom@gmail.com; 4 Grupo AFySE, Investigación en Actividad Física y Salud Escolar, Escuela de Pedagogía en Educación Física, Facultad de Educación, Universidad de Las Américas, Santiago 8370040, Chile; acastillop85@gmail.com; 5 Faculty of Physical Education, National Pedagogical University, Bogotá 110221, Colombia; babecerrap@pedagogica.edu.co; 6 Escuela de Ciencias de la Actividad Física, el Deporte y la Salud, Universidad de Santiago de Chile (USACH), Santiago 9170022, Chile; claudio.farias.v@usach.cl; 7 Facultad de Ciencias de la Vida, Universidad Viña del Mar, Viña del Mar 2520000, Chile; guillermo.cortes@uvm.cl; 8 Escuela de Ciencias del Deporte y Actividad Física, Facultad de Salud, Universidad Santo Tomás, Talca 3460000, Chile; mrivera3@santotomas.cl; 9 Vicerrectoría de Investigación e Innovación, Universidad Arturo Prat, Iquique 1110939, Chile; 10 Escuela de Odontología, Facultad de Odontología, Universidad Andres Bello, Concepción 3349001, Chile; 11 Faculty of Education and Social Sciences, Universidad Andres Bello, Viña del Mar 2520000, Chile; rodrigo.yanez.s@unab.cl; 12 School of Medicine, Universidad Espíritu Santo, Samborondón 0901952, Ecuador

**Keywords:** bibliometrics, physical fitness, athletic performance, biomechanical phenomena, sports medicine, exercise test, muscle strength, motor skills

## Abstract

Handgrip strength (HGS) has been considered as an indicator of muscle strength and overall physical fitness, with increasing relevance in sports science for talent identification and performance monitoring. However, no bibliometric study has been conducted to map the HGS research landscape in athletic contexts. A bibliometric analysis was conducted in the Web of Science Core Collection database, retrieving 229 publications. Typical bibliometric laws (i.e., Price’s, Bradford’s, Lotka’s, and Zipf’s) were employed to analyze publication trends, core journals, influential authors, country contributions, and keyword co-occurrences. Annual publications increased exponentially, especially after 2019, reaching 37 documents in 2024. *The Journal of Strength and Conditioning Research* and *Journal of Sports Medicine and Physical Fitness* were the most prominent journals. The United States and Spain led in productivity and impact. Key research themes included strength, performance, body composition, and physical fitness, with HGS demonstrating significant associations with sport tasks such as throwing, racquet sports, and weightlifting. HGS constitutes an accessible and valuable tool for assessing and predicting athletic performance, especially in sports requiring upper body strength and coordination. Future research should aim to expand database inclusion and address identified gaps, such as the relationship between HGS training and sport-specific outcomes.

## 1. Introduction

A strong body of evidence links muscle strength to overall health and physical fitness, demonstrating a characteristic decline with advancing age [1,2]. Thus, muscle strength has commonly been assessed using handgrip strength (HGS), as it relates to the expression of upper limb strength and its interaction with other muscle groups [3,4]. The assessment of HGS typically follows standardized dynamometry protocols that define key parameters such as body posture, number of trials, and hand dominance. The main associations that have been investigated are the relationship between HGS and aerobic capacity [5,6,7], physical fitness [8], and fitness level [9,10]. Models aimed at normalizing athlete performance based on body mass ratios have also been studied in strength sports [11,12]. Strong correlations exist between HGS and strength measurements from major muscle groups, establishing it as a representative marker of overall muscular capacity [13,14]. Some studies have reported that HGS is associated with athletic performance in racquet sports, specifically defining interactions between training models based on improving the tennis serve and HGS [15].

Studies in weightlifting have shown that HGS was associated with snatch performance [16,17]. In youth soccer athletes, it was determined that the integration of HGS and age collected up to 67% of the variance in sprint tests (10 and 30 m) and 61% in jump tests (i.e., Countermovement Jump, Abalakov Jump, Squat Jump), which indicates that HGS could be a relevant and practical predictor in specific soccer actions when combined with age [18]. Another cross-sectional study evaluating 1162 athletes (725 men and 437 women) concluded that HGS is significantly related to sex, specifically in women, and is a determining performance variable in ball and racket sports [19]. However, a study investigating the effect of anthropometric variables and HGS on the likelihood of competitive success in female judokas determined that HGS, Body Mass Index, and sitting height do not appear to be indicators of athletic performance [20]. A brief review study on HGS and athletic performance concluded that HGS is related to the specific movement patterns performed in sports, especially rotational movements that require the sum of forces and torques to generate sequences from large muscle groups to smaller muscle groups that allow angular velocities to be expressed with the hand as the end point of the movement [21]. Other studies have investigated the decline in HGS and its relationship with age in weightlifters competing in the 2022 Olympic Masters Championship (*n* = 168; 104 women and 64 men), demonstrating that the reduction in HGS progressively decreases in sports that demand power [17]. This evidence highlights the context-dependent nature of HGS as a performance indicator. Therefore, targeted improvements require careful manipulation of key factors—(i) the assessment/training protocol, (ii) suspension time, (iii) intensity, and (iv) workload—to ensure sport-specific relevance and efficacy [22].

HGS is a crucial determinant of performance in various sports requiring upper limb force and hand–eye coordination, including baseball, climbing, golf, wrestling, tennis, and swimming [21]. Research studies have established significant correlations between HGS and morphological indicators like hand size, finger length, and forearm circumference [23,24]. These findings underscore the relevance of HGS for athletes and coaches, highlighting the need to understand its impact on sport-specific actions requiring speed, precision, and strength [25].

While the physiological and performance relationship of HGS is increasingly studied, the literature remains fragmented over different sports, athlete levels, and methodological approaches. To consolidate this knowledge and guide future research, a systematic mapping of the scientific landscape is essential [26]. Bibliometric analysis is a powerful technique for identifying research trends, key contributions, and underexplored fields [27,28]. However, to our knowledge, no bibliometric study has yet been conducted to visualize and synthesize the research landscape concerning HGS and sports performance.

Therefore, this study aimed to perform a bibliometric analysis to map publication trends, identify thematic emphases, and highlight research gaps in the scientific literature concerning handgrip strength (HGS) and sports performance, offering a comprehensive overview to guide future research.

## 2. Materials and Methods

### 2.1. Study Design

A bibliometric analysis was conducted using typical bibliometric laws [29,30,31,32,33,34]. For this analysis, the Web of Science Core Collection (WoSCC) was consulted, which is particularly useful for researchers seeking to evaluate the impact of their work.

Web of Science (WoS) provides researchers with various functions, such as meta-analysis and citation trend tracking, ensuring high data integrity by excluding self-citations and duplicates. These features make WoS an indispensable tool for identifying influential articles, exploring emerging research areas, and conducting bibliometric studies [35]. WoSCC is a comprehensive database that supports the identification of research trends and offers a wealth of bibliometric data, contributing to its broad usage for scholarly research [35].

### 2.2. Search Strategy

An advanced search was conducted in the WoSCC, selecting the following databases: Citation Index Expanded (SCIE), Social Science Citation Index (SSCI), and Emerging Sources Citation Index (ESCI). The search was performed on 25 July 2025. To enhance efficiency and accuracy, the following search query was used:

(TI = ((“handgrip strength” OR “grip strength” OR “grip force” OR “grip dynamometry” OR “isometric hand strength”) AND (“sport *”) AND (“performance *”))) OR AB = ((“handgrip strength” OR “grip strength” OR “grip force” OR “grip dynamometry” OR “isometric hand strength”) AND (“sport *”) AND (“performance *”)). The TI tag indicates that the search will be conducted in the title, while the AB tag indicates that the search will be performed in the abstract.

The following inclusion criteria were applied: (1) peer-reviewed articles or reviews indexed in the WoSCC to capture the structure, influence, and knowledge consolidation of a research field; (2) studies addressing handgrip strength in sport or athletic performance contexts; and (3) studies involving athlete populations. Exclusion criteria were as follows: (1) studies on clinical or non-athletic populations; (2) animal studies; and (3) documents unrelated to the study topic (HGS, performance, and sport). No limitations were applied regarding the publication date or language.

### 2.3. Data Retrieval and Analysis

In order to reduce potential bias during the filtering and selection process, we used the PRISMA method as a transparency tool for document identification and selection [36]. The search was conducted independently by researchers D.S.-G. and A.C.-P., ensuring consistency in the documents retrieved. The titles and abstracts of the identified publications were reviewed to confirm that they met the established inclusion criteria.

To assess the scientific status and current trend in academic production, the annual publication trend was analyzed using the exponential growth model commonly referred to as Price’s law, as further developed by Dobrov, which describes cumulative growth patterns in scientific research [29,31]. An exponential regression model was fitted to the annual publication counts, and the coefficient of determination (R^2^) was calculated as an indicator of model fit, allowing for evaluation of the extent to which publication output followed an exponential growth pattern over time [37]. The analysis covered from 2011 to 2024. The start date was set to 2011 following a preliminary review that identified it as the year when sustained and relevant publication activity on the topic began. Microsoft Excel was used both for calculating R^2^ and for graphical representation of the results. Subsequently, a descriptive analysis was conducted of the WoS’s thematic categories under which the documents were classified.

To identify highly cited publications within the scientific literature, the H-index was used as a bibliometric indicator of citation impact [30]. Publications were sorted in descending order based on the number of citations obtained in WoS. This allowed for determining the cutoff point, selecting the document “h” with “h” citations or more. A graph was created using Microsoft Excel to show the distribution of documents and citations, which helped establish the cutoff point for the H-index.

To identify the journals with the highest number of publications on the topic, Bradford’s Law of Concentration of Science was applied [33,38]. Using this law, the core journals were established, referring to the journals with the largest number of publications, and these fell within the top three. This approach helped us understand how scientific articles in this field are distributed across different journals. Following this, a descriptive analysis was conducted on these core journals, covering aspects such as journal name, publisher, number of documents, citations, normalized citations (citations per document), Journal Impact Factor (JIF), quartile ranking, and the percentage of Gold Open Access content.

Subsequently, Lotka’s law was applied to estimate the number of most productive authors, calculated as the square root of the total number of authors [32]. Previously, the authors were standardized by eliminating duplicates. The correct application of this law was verified by discrete counting of the number of articles per coauthor and coauthors by level of production. Coauthorship networks were also elaborated with the VOSviewer version 1.6.20 (Centre for Science and Technology Studies, Leiden University, The Netherlands), considering the identified authors and their average publication years [39]. Those coauthors who had one or more papers among the most cited were classified as prominent. Finally, a descriptive analysis of these authors was carried out, detailing their names, number of publications, citations received, number of highly cited articles, and the color of the production clusters to which they belonged.

A descriptive assessment of coauthoring countries was made by examining the number of articles per country. A graph was made in Microsoft Excel to show the relationship between the collaborating countries and their total citation counts. To identify the author keywords with the highest occurrence, Zipf’s law was applied. Co-occurrence analysis was conducted in VOSviewer version 1.6.20 (Centre for Science and Technology Studies, Leiden University, Leiden, The Netherlands), allowing for the detection of thematic clusters and the determination of the average publication year for each term [34].

## 3. Results

**Figure 1:**
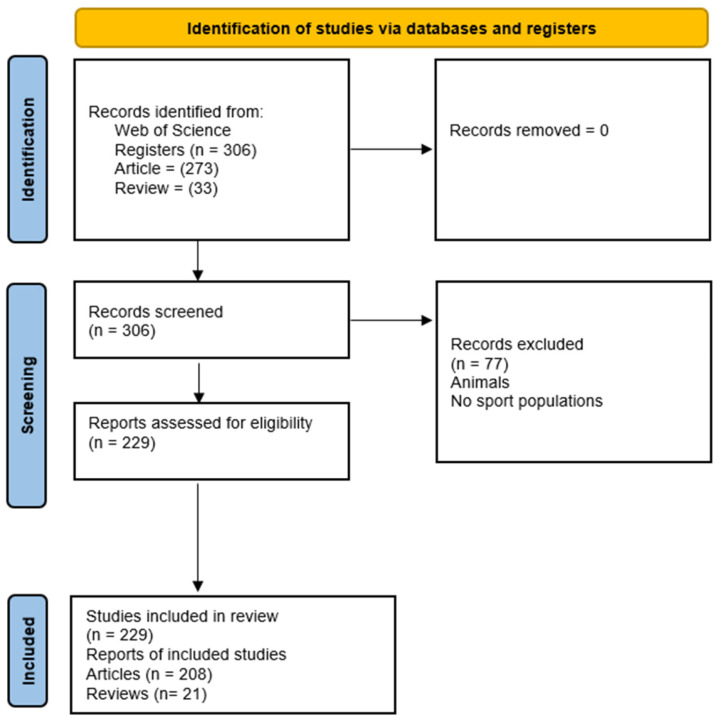
Flowchart of the selection process.

After the search, 306 documents were retrieved from WoS. Once the screening was completed, 229 documents met the inclusion criteria and were finally included in the analysis (208 articles and 21 reviews). Figure 1 shows the flowchart of the screening process.

### 3.1. Annual Publications Trends on Handgrip Strength and Performance in Sport-Related Research

The analysis of annual publications indicates an increasing volume of scientific research addressing HGS in sport performance contexts over time. The earliest record identified in the database dates back to 1985; however, the bibliometric analysis revealed no continuity in publication output prior to 2009, and therefore quantitative analyses were restricted to the period 2011–2024.

**Figure 2:**
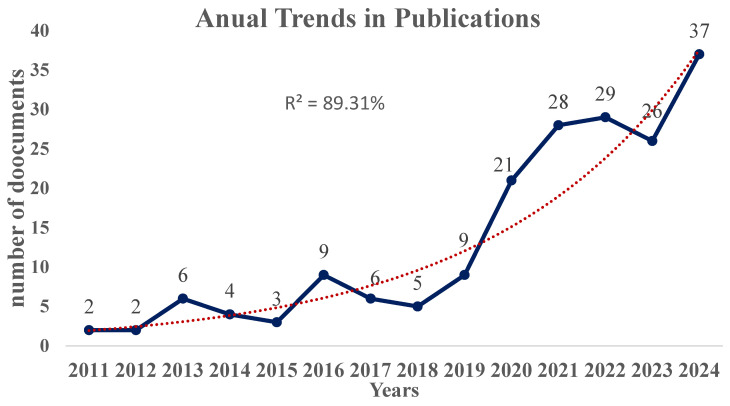
Annual publication trends in research on handgrip strength and sport performance (data source: Web of Science Core Collection). The blue points represent the yearly publication frequency, while the red dotted line shows the exponential growth trend.

As illustrated in Figure 2, the number of publications remained relatively low and inconsistent between 2011 and 2018, with fewer than 10 publications per year. From 2019 onwards, the scientific literature exhibits a marked increase in annual output, with the annual output more than quadrupling between 2018 (5 documents) and 2021 (28 documents).

Despite a slight drop in 2023 (26 documents), the field of research recovered quickly, reaching a record high of 37 publications in 2024. This pattern suggests an overall growth trend in scientific production within the literature, as identified by the fitted exponential trendline (R^2^ = 89%).

### 3.2. WoS Categories

In recent years, the scientific literature addressing HGS and sport performance has been disseminated across multiple disciplinary domains, as reflected in its association with multiple Web of Science (WoS) thematic categories.

Among the 51 identified categories, Sport Sciences was the most frequent, with a total of 117 documents. Other significant categories include Nutrition and Dietetics (21 documents), and Hospitality, Leisure, Sport, and Tourism (18 documents). Additionally, the categories Public, Environmental, and Occupational Health (11 documents) and Rehabilitation (11 documents) also contributed to the field.

### 3.3. Most Cited Documents

**Figure 3:**
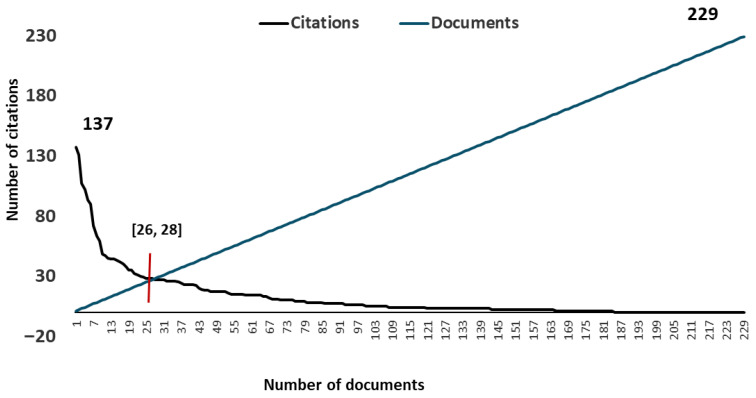
H-index distribution of documents within the literature. The cut-off point is 28 citations, the requirement for a paper to be considered among the most cited (data source: Web of Science Core Collection). The black line represents the citation frequency, and the blue line represents the paper count. The figure was generated using Microsoft Excel.

The H-index was calculated to identify the most influential documents within the scientific literature on HGS and performance in the sport context. Of the total documents published (229 papers), the H-index was determined to be 26, meaning that 26 documents received at least 26 citations. This cut-off point ([26 documents, 28 citations]) was used to classify the most cited works within the literature (Figure 3). The complete list of the 26 most cited papers can be found in the Supplementary Materials (File S1: Annex I. Most Cited Papers).

### 3.4. Journals

When the journals were grouped by terciles, according to the level of production, it was observed that inclusion in the first tercile required a minimum of four or more publications. However, journals with four or more papers accumulated 38%, exceeding 33.3%. On the other hand, journals with five or more accumulated 28%, within the first tercile. The most restrictive option was chosen, and the zones were distributed as shown in Table 1. The Nucleus Zone consists of eight journals (7% of all journals), which published 65 documents (28.3% of total publications). Zone I includes 35 journals (30%) with 90 documents (39.3% of publications). Finally, Zone II includes 74 journals (63%), which published 74 documents (32.3%). Table 1 also shows the Bradford zones, how the documents and journals were distributed within them, and the adjustment of this distribution to the theoretical Bradford estimate (% Error = −1.0%).

Table 2 summarizes the core journals in which the scientific literature on HGS and sport performance is concentrated.

### 3.5. Prolific and Prominent Authors

A total of 1183 coauthors published articles. These coauthors published between one and six articles. Applying Lotka’s law, given a total of 1183 authors, this yielded an expected value of approximately 34 prolific authors (the square root of 1183). The analysis identified 69 authors with two or more publications, classified as prolific authors within the literature.

**Figure 4:**
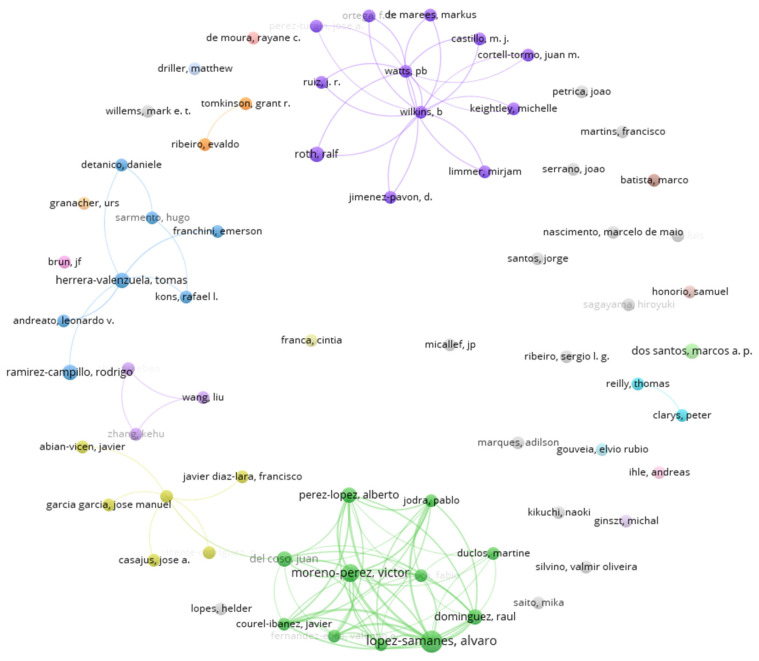
Prolific coauthor graph. Coauthorship network of prolific authors in handgrip strength research within sports performance contexts. Each node represents an author, and the node size reflects the number of publications within the dataset. Links indicate coauthorship relationships between authors, with thicker links representing stronger collaborative ties. Colors de-note clusters of authors identified through coauthorship network analysis using a community detection algorithm implemented in VOSviewer version 1.6.20.

In contrast, the bibliometric analysis found a high volume of coauthors with one (1114 coauthors) or two papers (60 coauthors) (Figure S1). Figure 4 shows the 69 prolific coauthors and the production network they form when performing a coauthorship analysis. The complete list of prominent authors can be found in Table 3.

### 3.6. Countries

**Figure 5:**
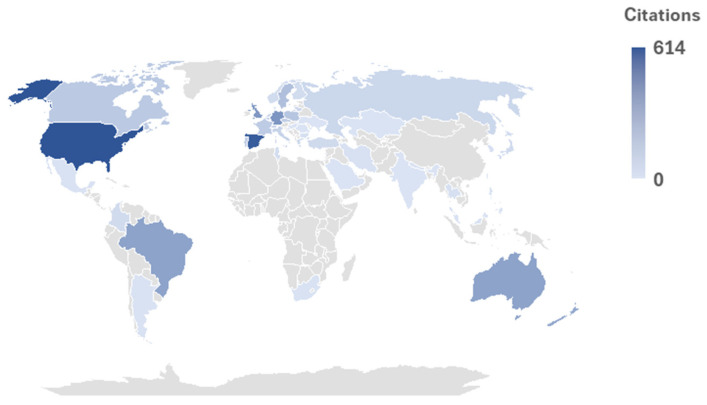
Geographical distribution of publications according to number of citations (data source: Web of Science Core Collection).

As shown in the analysis, the United States is the leading country in terms of citations (614 citations and 30 papers). Spain is the second country with 581 citations (35 documents). Other significant contributors include Germany (19 papers, 337 citations), the United Kingdom (22 papers, 324 citations), and Brazil (26 papers, 277 citations). Countries such as Australia (14 papers, 277 citations), New Zealand (6 papers, 254 citations), and Sweden (5 papers, 152 citations) also show remarkable research on this topic. Figure 5 shows a global map highlighting the countries involved in the development of this topic of study.

### 3.7. Keywords Analysis

**Figure 6:**
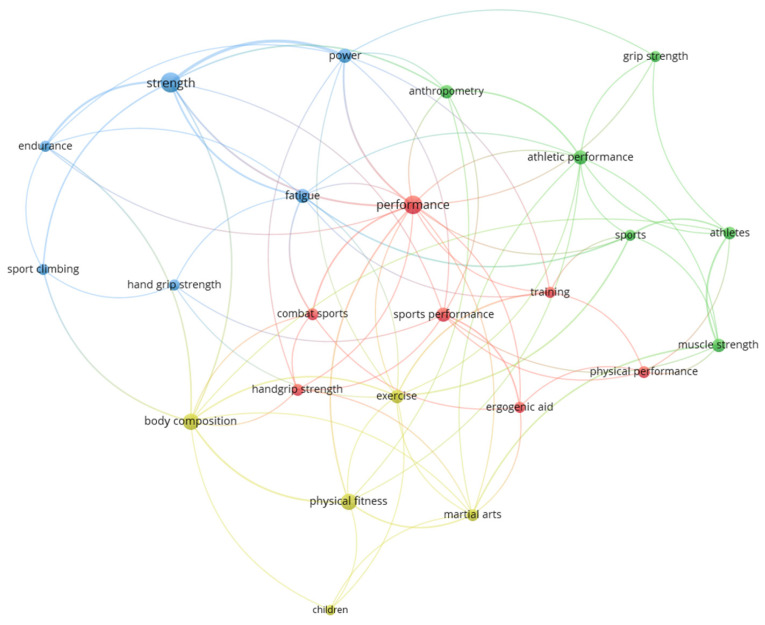
The most used author keywords graph. Each node represents an author keyword, with node size proportional to its frequency of occurrence in the dataset. Links indicate co-occurrence relationships between keywords, with thicker links representing stronger associations. The figure was generated using VOSviewer version 1.6.20.

Following Zipf’s law, of a total of 674 author keywords, the analysis identified 24 keywords with 6 or more occurrences, representing the most relevant concepts in the research on HGS and performance in the sport context. Figure 6 shows the 24 most used author keywords, as well as their grouping into thematic clusters. Handgrip strength, sport performance, handgrip strength, or physical fitness were the terms with the most current average year of publication (Figure S3).

### 3.8. Synthesis of Research Applications and Trends

The bibliometric indicators outline the evolving research landscape. The primary application of HGS, evidenced by the Sport Sciences category and core journals, is as a practical tool for athlete monitoring, talent identification, and strength assessment. The keyword analysis reveals that research has primarily investigated its relationship with broad constructs like strength, performance, body composition, and physical fitness. The exponential growth post-2019, concentrated in journals focused on strength conditioning and sports medicine, marks a clear trend: the transition of HGS from a general health marker to a specialized sports performance biomarker. The prominence of authors and countries with strong traditions in sports science further solidifies this trend. A notable finding for future directions is the relative scarcity of the specific term “handgrip strength” among the most frequent keywords, suggesting that research is often embedded within broader thematic studies. This highlights an opportunity for more targeted, sport-specific HGS research and the need for standardized keyword usage to improve research discoverability.

## 4. Discussion

As the first bibliometric study mapping the publications on the subject of HGS within the sports performance context, this analysis provides a global overview of the evolution in the field. The analysis of 229 documents from the WoS’s main collections revealed an accelerated growth over the past decade, interpreted as a recognition of HGS as a critical biomarker of athletic skill, moving beyond its traditional health assessment role into the field of talent identification, performance prediction, and training monitoring in sports science.

### 4.1. Annual Publications Trends

This bibliometric study reveals a sustained increase in scientific production related to HGS and its relationship to athletic performance, particularly since 2019, reaching a peak of 37 publications in 2024. This growth coincides with the consolidation of HGS as a robust and reliable marker of upper limb muscle strength, as well as its association with health and fitness indicators throughout life [1,14,40]. The growing body of literature on this topic reflects the trend toward considering HGS not only as a clinical parameter but also as a functional predictor in sports contexts [17] and as a valuable and efficient resource for performance assessment and talent development [18].

### 4.2. Categories, Most Cited Documents, and Journals

The analysis of WoS categories and core journals directly addresses our aim to define the primary applications and trends in HGS research. The dominance of the Sport Sciences category, followed by Nutrition and Dietetics, Hospitality, Leisure, and Sport and Tourism, underscores the fact that HGS is mainly investigated as a functional performance and athlete health biomarker, with strong cross-disciplinary links to nutritional status and applied sport [14,41].

Identifying the most cited documents is crucial to understanding the essential knowledge and major trends that have influenced the field. The two most cited works, a review establishing the biomechanical role of HGS in sports [21] and a large-scale study on secular fitness trends [42], highlight that the intellectual foundation rests on integrative physiological frameworks and population normative assessments. This indicates a trend toward synthesizing mechanistic understanding with practical monitoring tools.

Furthermore, the concentration of publications in a core of journals, led by the *Journal of Strength and Conditioning Research* and the *Journal of Sports Medicine and Physical Fitness*, indicates that the primary distribution channel for this topic is oriented towards strength conditioning, physical fitness, and clinical sports medicine. This journal profile aligns with the field’s focus on practical assessment and athlete monitoring, informing practical recommendations for coaches and practitioners.

### 4.3. Prolific and Prominent Authors, Countries, and Keywords Analysis

The analysis of coauthorships and the main bibliographic sources reveals an expanding scientific field, with key works such as the widely cited (137 citations) review [21], which establishes an integrative framework for understanding the biomechanical and functional role of HGS in different sports. Furthermore, the recognition of prominent authors facilitates the consolidation of collaborative networks and the focus on emerging lines of research, which is essential for progress given the heterogeneity of sporting contexts and assessment methods. From a practical perspective, HGS measurement is presented as a useful and low-cost tool for monitoring the strength and physical condition of athletes.

The most productive countries were the United States and Spain, with the highest number of citations and documents. This may be related to the fact that these countries are among the leaders in research [43], as well as, in the case of the USA, one of the countries with the greatest contribution to sports science [44]. Finally, regarding the use of keywords, it was expected that HGS would be used more frequently; however, keywords such as “strength,” “performance,” “body composition,” and “physical fitness” were the most commonly used. This relationship may be attributed to the established association between muscle mass, function, and strength with fundamental health parameters that are universally relevant [40], and, in the case of athletes, if they use their hands, it further promotes the development of their HGS [45]. However, it is worth emphasizing that proper keyword usage enables precise identification of studies and their content, making it a critical component of effective research [46].

Contrary to the previously noted trend, one study found that HGS, Body Mass Index, and sitting height did not predict the probability of success in competitive judo [20]. This underscores the need to interpret HGS within a specific context, according to the discipline and the characteristics of each athlete. Several studies highlight the relevance of HGS for describing and predicting performance in disciplines that require strength, power, and visuomotor coordination [15]. For example, HGS directly influences tennis serve performance, underscoring the relationship between strength and sport-specific technique [15]. In strength sports, such as weightlifting, GS correlates with performance in the snatch phase and is an integral component of muscle power profiles [16,17]. Further supporting its role as a marker of athletic proficiency, a study on Brazilian jiu-jitsu athletes found that experts demonstrated significantly greater HGS than novices, directly linking HGS to a higher level of skill and training experience [47]. Similarly, in youth soccer, integrating HGS with variables such as age explained up to 67% of the variability in sprint and jump tests [18]. Supporting its broad applicability in talent development, comprehensive percentile references for HGS in young elite athletes have been established, demonstrating consistent increases with maturation and chronological age and highlighting its utility as a practical benchmark for identification and monitoring [48]. This highlights the potential of this metric as a predictive variable in specific sports activities and its easy accessibility for coaches.

Regarding the anthropometric factors that modulate HGS, previous studies [23,24] have confirmed that hand dimensions, finger length and circumference, and forearm circumference are fundamental determinants, indicating that morphology should be considered for both the assessment and interpretation of HGS in athletes. This anthropometric relationship is particularly relevant in sports that demand hand–eye coordination and speed of action, such as handball [25], where the force exerted is integrated with complex neuromuscular patterns.

### 4.4. Strengths and Limitations

The interpretation of these findings should consider both the strengths and limitations of this study. In terms of strengths, as the first bibliometric study on this topic, this study provides an overview of scientific output, WoS categories, most cited documents, journals, most prolific and prominent authors, countries, and most frequently used keywords. Similarly, this work offers a general overview, which allows for informed decision-making regarding scientific output, categories, researchers, and countries that conduct the most research on this topic. Regarding its limitations, this study only considered the use of one database. Although it represents one of the most used databases widely adopted in bibliometric research, it may exclude relevant studies not indexed within its repository, potentially introducing a selection bias. Another limitation of this study is the absence of an analysis into device standardization and usage protocols, making it impossible to formulate guidelines for its use. Additionally, athletes’ competitive level (e.g., elite vs. sub-elite) was not systematically coded or analyzed. Consequently, the bibliometric patterns identified in this study reflect the research field in an aggregated manner and may mask differences in publication trends, thematic emphases, or research focus between elite and sub-elite athlete populations. Future bibliometric studies could address these limitations. Consequently, these limitations should be considered when generalizing the findings, as the mapped landscape reflects the indexed literature within a specific database and does not account for the variability in practical measurement methodologies.

### 4.5. Practical Applications and Future Research

This research provided an overview of HGS utilization, highlighting the current and emerging research direction in this field. HGS serves as a practical and low-cost tool for the monitoring of athletic development. The consistent relationship between HGS and sport performance (e.g., throwing velocity, racket sports proficiency) supports its use as a diagnostic tool for the evaluation of training interventions and decisions after upper extremity injuries. The findings of this bibliometric analysis indicate that HGS measurement is particularly suitable, and its utility is most evidenced for sports where the hand is a primary point of force application, control, or grappling. This includes strength and power sports such as weightlifting [16,17] and wrestling [20], racket sports like tennis [15], throwing events in athletics, climbing [22], and combat sports such as judo [20] and Brazilian Jiu-Jitsu [20]. Sports organizations in those disciplines can integrate HGS assessments into regular testing batteries to track athlete progress and identify deviations from expected growth trajectories.

Following this logic, future lines of research should prioritize an in-depth analysis of both mechanical or digital assessment devices, as well as incorporate additional scientific databases. Investigation is needed to explore how HGS interfaces with sports performance indicators (e.g., grip endurance in climbing, asymmetric strength in throwing events) to develop event-specific assessment protocols. Future works should also explore the relationship between HGS and other physiological systems (e.g., endocrine responses, cognitive function) to better understand its role as a biomarker of overall athletic readiness and health status.

## 5. Conclusions

Research related to HGS has experienced sustained growth over the past years, especially after 2019, when the number of related papers began to increase suddenly. This increase indicates that HGS is becoming more widely acknowledged as a straightforward and useful measure of performance in athletes. This review of the available literature makes it clear that the topic has captured the attention of sports scientists, clinicians, and exercise physiologists interested in quantifying upper limb function using this accessible and inexpensive measure. From a practical point of view, the evidence presented can confirm that the HGS is a valuable tool for both predicting and monitoring athletic performance. Its predictive utility has been mainly observed in sports where strength, power, and complex coordination are fundamental, such as tennis, weightlifting, and combat sports.

It is important to note that when additional factors, such as age and other anthropometric measurements, are taken into account, the HGS explanatory power tends to rise, improving predictive function for certain tasks like jumping or running. However, it is crucial to remember that there is not a uniform correlation between HGS and performance. As a result, its influence depends on the particular requirements of each activity and the characteristics of each athlete. The findings of this bibliometric review offer direct practical implications. For researchers, they highlight the necessity to standardize assessment protocols and investigate causal training effects. For coaches and sports practitioners, they reinforce the value of HGS as a low-cost, efficient tool for athlete monitoring and talent identification in relevant sports, provided it is interpreted within the specific sport context. This review offers a glimpse into some prospective research directions. Establishing normative data and comparing research would be made easier with the creation of standardized assessment processes. Additionally, even while correlations are seen, further research is required to examine the causal pathways connecting particular HGS training to gains in sports performance.

## Figures and Tables

**Table 1:** Bradford’s zones for handgrip strength and performance in sport-related research.

Zone	Number of Documents on Thirds (%)		Journals (%)		Bradford Multipliers	Journals (Theoretical Series)	
Nucleus	65	28.3%	8	7%		16 × (n0)	8
Zone I	90	39.3%	35	30%	4.4	16 × (n1)	26
Zone II	74	32.3%	74	63%	2.1	16 × (n2)	84
TOTAL	229	100%	117	100%	3.2		118
						% Error	−1.0%

**Table 2:** Core journals for handgrip strength and performance in sport-related research.

Publication Titles	Doc.	Cit.	Norm. Cit.	JIF	Quartile	% O.A.
*Journal of Strength and Conditioning Research*	15	380	25	3	Q1	2.9
*Journal of Sports Medicine and Physical Fitness*	13	112	9	1.3	Q3	3.9
*Journal of Science and Medicine in Sport*	7	237	34	3.4	Q1	30.2
*Nutrients*	7	124	18	5	Q1	100.0
*European Journal of Sport Science*	6	258	43	3	Q1	0.0
*Archives of Budo*	6	114	19	1.5	Q3	58.8
*Retos-Nuevas Tendencias en Educacion Fisica Deporte y Recreacion*	6	27	5	1.2	Q3	29.7
*Sports*	5	14	3	2.9	Q1	100.0

Doc. (number of documents); Cit. (citations); Norm. Cit. (normalized citations: citations/documents); JIF (Journal Impact Factor); quartile (JIF quartile); % O.A. (percentage of open access).

**Table 3:** Prominent coauthors.

Author’s Name	Cluster	Doc.	Cit.	Most Cited Papers
Del Coso, Juan	Green	3	115	2
Abian-Vicen, Javier	Yellow	2	110	2
Javier Diaz-Lara, Francisco	Yellow	2	110	2
Watts, Pb	Purple	2	209	2
Wilkins, B	Purple	2	209	2
Lopez-Samanes, Alvaro	Green	6	87	1
Moreno-Perez, Victor	Green	4	76	1
Dominguez, Raul	Green	3	45	1
Perez-Lopez, Alberto	Green	3	48	1
Brun, Jf	Pink	2	19	1
Casajus, Jose A.	Yellow	2	163	1
Castillo, M.J.	Purple	2	143	1
Franchini, Emerson	Blue	2	63	1
Garcia Garcia, Jose Manuel	Yellow	2	110	1
Glenn, Jordan M.	Yellow	2	68	1
Granacher, Urs	orange	2	37	1
Jimenez-Pavon, D.	Purple	2	143	1
Nakamura, Fabio	Green	2	33	1
Ortega, F.B.	Purple	2	143	1
Ribeiro, Evaldo	Orange	2	50	1
Ruiz, J.R.	Purple	2	143	1
Tomkinson, Grant R.	Orange	2	57	1
Vicente-Rodriguez, G.	Yellow	2	163	1

Doc. (number of documents); Cit. (citations); Clusters represent communities of authors identified through coauthorship network analysis, in which authors co-publish more frequently with each other than with authors in other clusters, as determined by a community detection algorithm implemented in VOSviewer version 1.6.20. Cluster colors are used for visualization purposes only.

## Data Availability

All data generated or analyzed during this study are included in this published article and its Supplementary Information files.

## Supplementary Materials

The following supporting information can be downloaded at https://www.mdpi.com/article/10.3390/sports14030101/s1, File S1: Anexe I. Most Cited Papers; Figure S1: Coauthors frequency publications; Figure S2: Coauthors years; Figure S3: Keywords average year of publication. References [49,50,51,52,53,54,55,56,57,58,59,60,61,62,63,64,65,66,67,68,69] are cited in the Supplementary Materials.

## Abbreviations

The following abbreviations are used in this manuscript:

ESCI	Emerging sources citation index
HGS	Handgrip strength
PRISMA	Preferred reporting items for systematic reviews and meta-analyses
SCIE	Science Citation Index Expanded
WoS	Web of Science
WoSCC	Web of Science Core Collection
