# Supplementary figures and images for: Mapping Handgrip Strength Research in Sports Performance: A Bibliometric Review of Applications, Trends, and Future Directions
Source: Sports (Basel). 2026 Mar 4;14(3):101. doi: 10.3390/sports14030101 (PMC13030412; doi:10.3390/sports14030101)

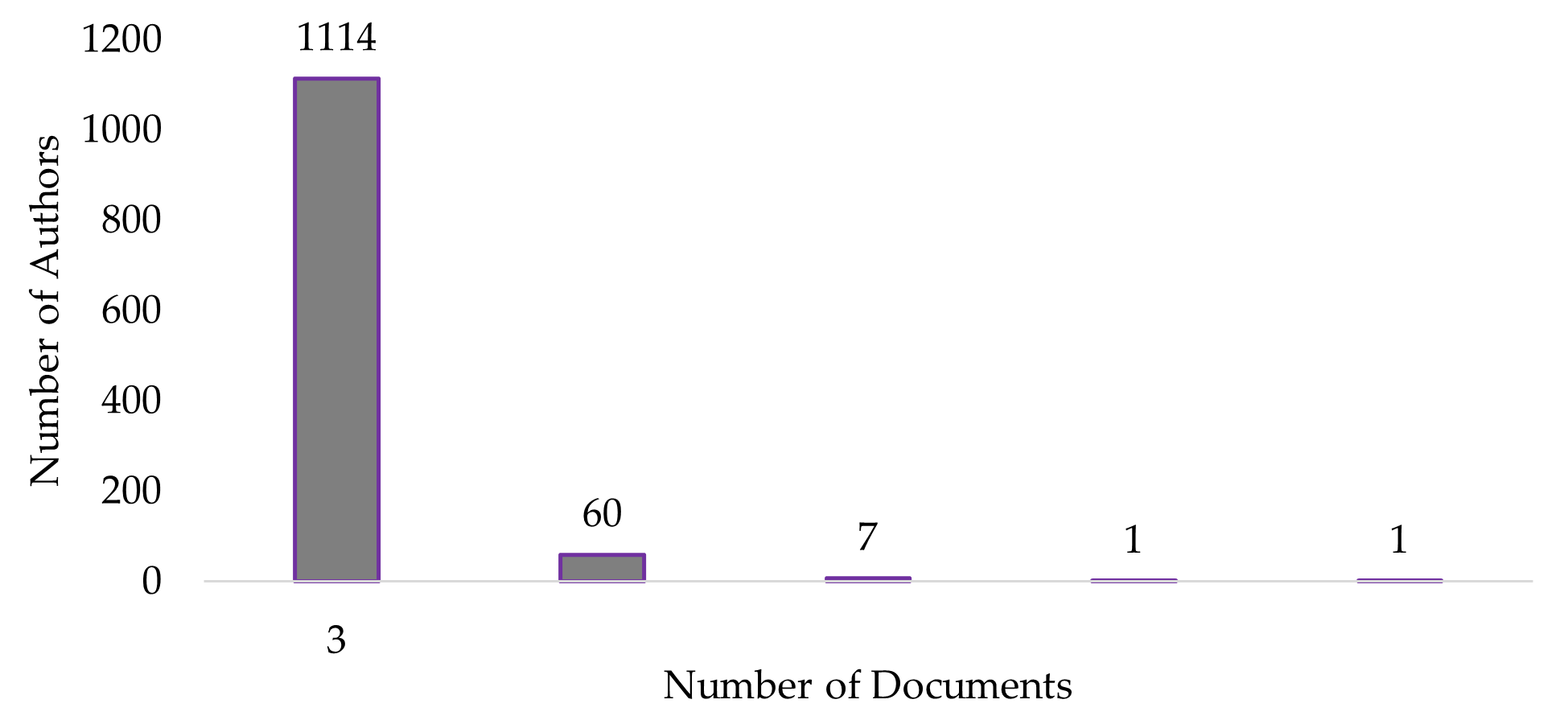

Supplement: Supplementary file 1 [file sports-14-00101-s001.zip › Figure S1 Coauthors frequency publications.png]

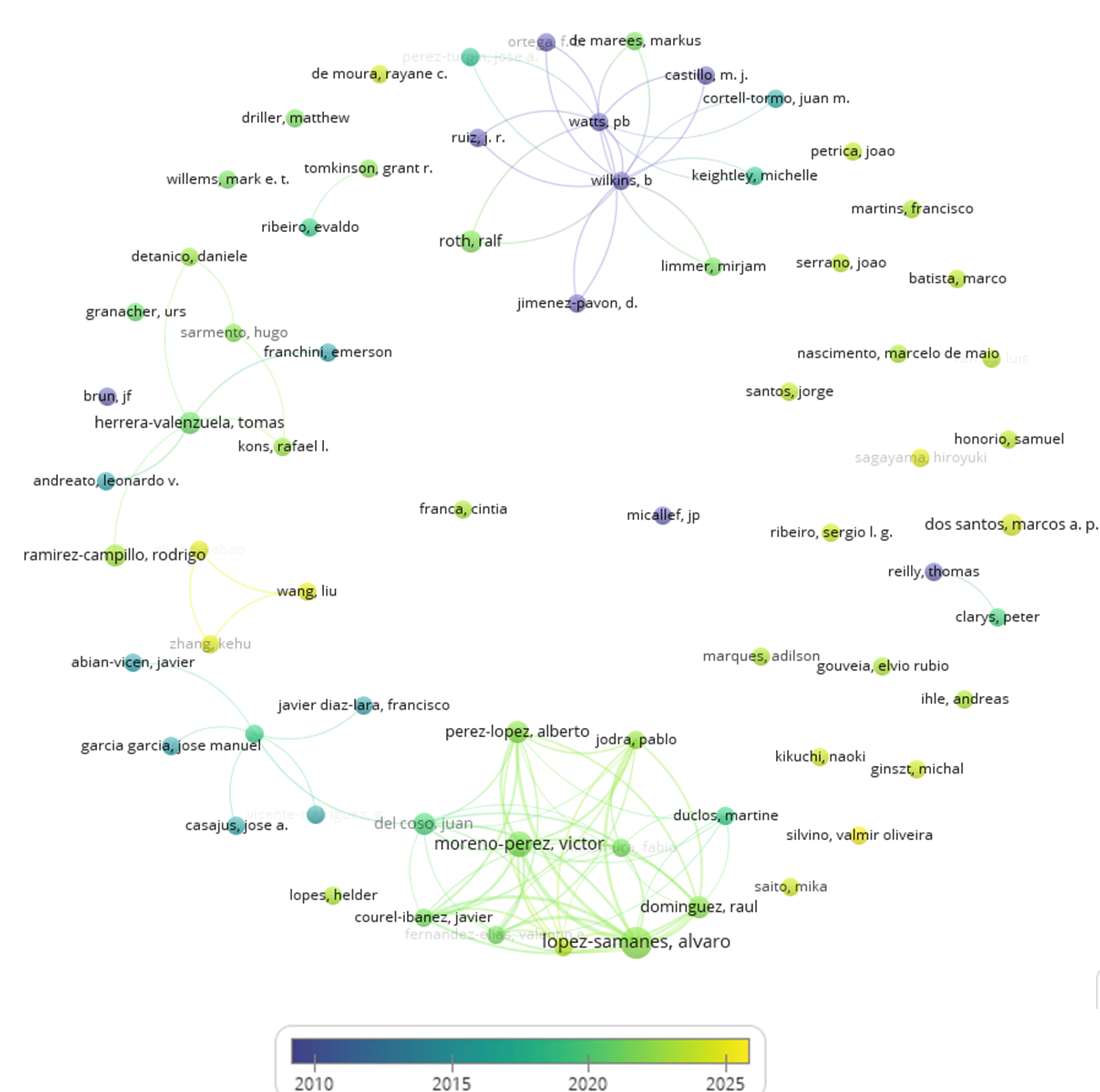

Supplement: Supplementary file 1 [file sports-14-00101-s001.zip › Figure S2 . Coauthors years.png]

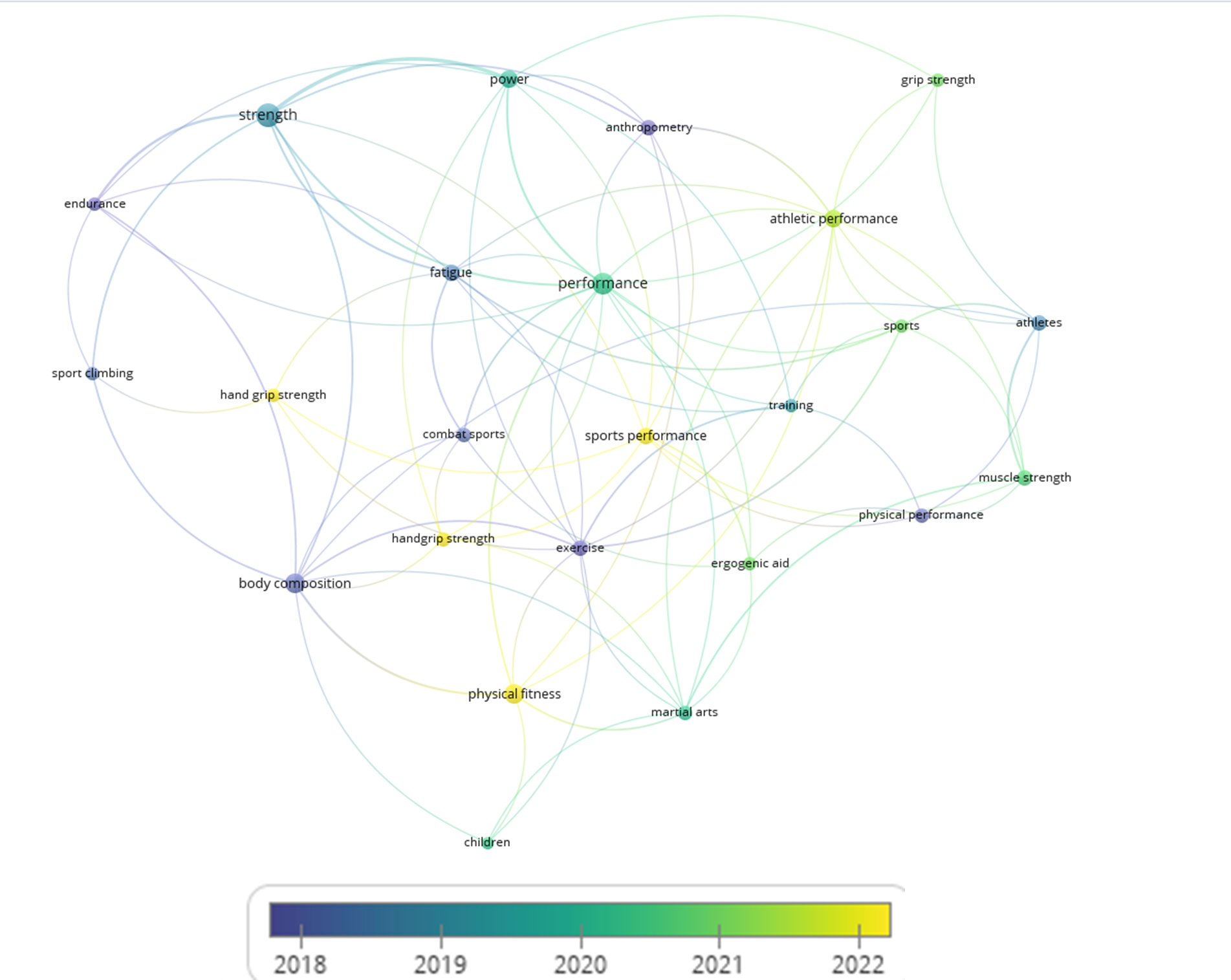

Supplement: Supplementary file 1 [file sports-14-00101-s001.zip › Figure S3. Keywords average year of publication.png]
